# Supplementary figures and images for: A regulatory module controlling stress-induced cell cycle arrest in Arabidopsis
Source: eLife. 2019 Apr 4;8:e43944. doi: 10.7554/eLife.43944 (PMC6449083; doi:10.7554/eLife.43944)

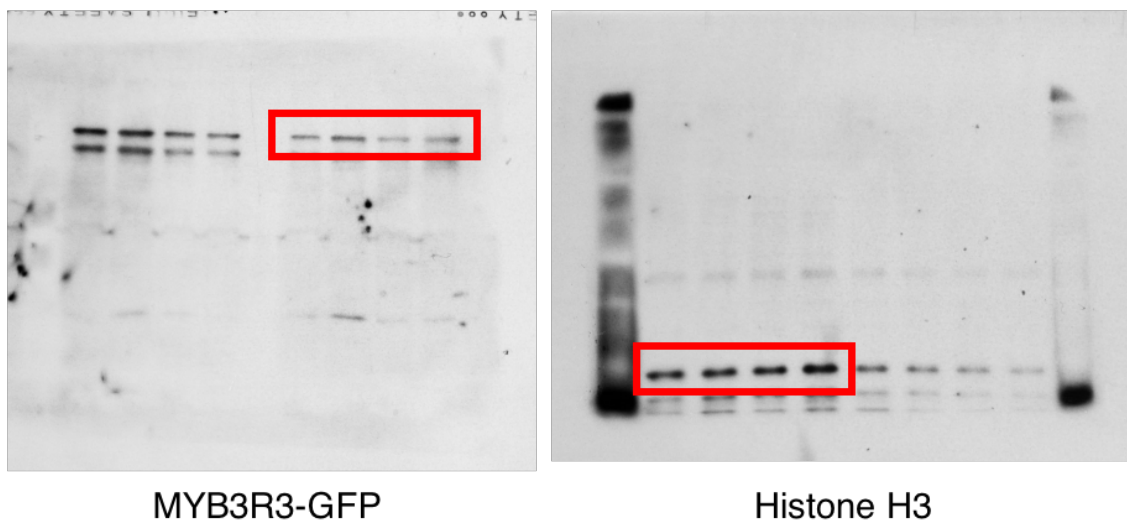

**Figure 6-source data 2.** Uncut blots.

The red sections mark blot results shown in Figure 6D.

Supplement: Figure 6—source data 2. [file elife-43944-fig6-data2.pdf]
