# Supplementary material for: A regulatory module controlling stress-induced cell cycle arrest in Arabidopsis
Source: eLife. 2019 Apr 4;8:e43944. doi: 10.7554/eLife.43944 (PMC6449083; doi:10.7554/eLife.43944)
Supplement: Figure 9—source data 2. [file elife-43944-fig9-data2.pdf]

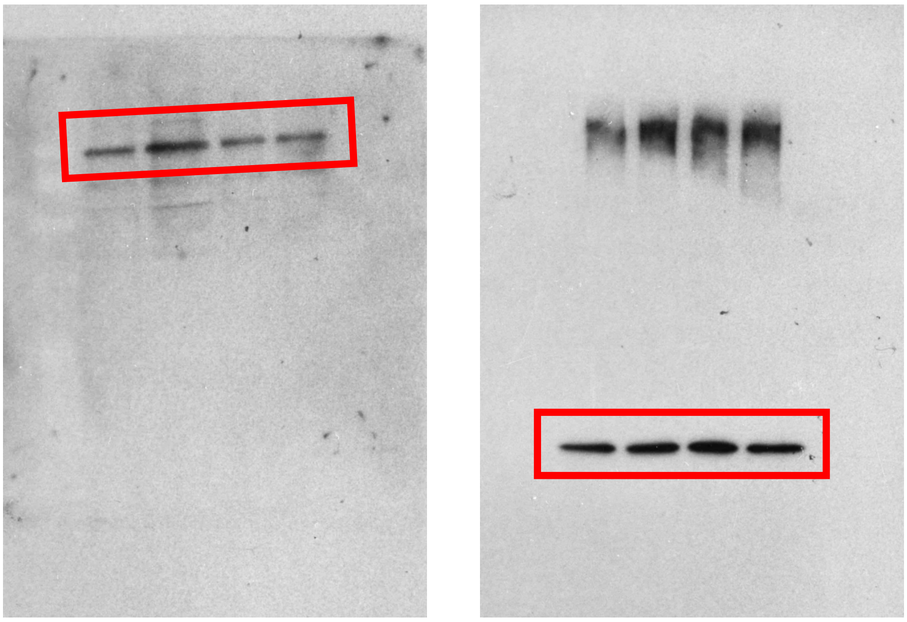

MYB3R3-GFP Histone H3

Figure 9-source data 2. Uncut blots.

The red sections mark blot results shown in Figure 9C.
