# Supplementary material for: A regulatory module controlling stress-induced cell cycle arrest in Arabidopsis
Source: eLife. 2019 Apr 4;8:e43944. doi: 10.7554/eLife.43944 (PMC6449083; doi:10.7554/eLife.43944)
Supplement: Supplementary file 1. [file elife-43944-supp1.docx]

**Supplementary File 1. Primers used for cloning, qRT-PCR, ChIP-qPCR and semi-quantitative RT-PCR**

**Cloning**

| *ANAC044*  promoter | 5’-AAAAAGCAGGCTTCCCACAGAAGATGACTTGAAGACC-3’  5’-AGAAAGCTGGGTTGATTCCCAAAACAGAGAGAGGGG-3’ |
| --- | --- |
| *ANAC085*  promoter | 5’-AAAAAGCAGGCTTCCTAATATTGGACACGCAAGTGAC-3’  5’-AGAAAGCTGGGTTCGGTTTAGTGTCAATAGCAAAGC-3’ |

**qRT-PCR**

| *ACTIN2* | 5’-CTGGATCGGTGGTTCCATTC-3’  5’-CCTGGACCTGCCTCATCATAC-3’ |
| --- | --- |
| *ANAC044* | 5’-GAGCGCTAGAAAGGGAACGA-3’  5’-CCCCGGAACTACTCTCACCTTC-3’ |
| *ANAC085* | 5’-AGCACACCGAAAACTAGTAC-3’  5’-CTTCAATAACACTCACATTCCC-3’ |
| *KN* | 5’-CTAATCAGAAGAGTGAAAAAGATG-3’  5’-TTCGTAGAAGCCATCTCAAGATC-3’ |
| *CYCB1;2* | 5’-ATTACGACACCTTGACGTTCTGTC-3’  5’-TTTGAGCAGTCCATAATCTCAGAC-3’ |
| *EHD2* | 5’-CCTGAACACACCAGAGGTCG-3’  5’-CGTTTATGGGTTTGTCATTG-3’ |
| *PLE* | 5’-CCAAGCTCCCAGGTATGGTTGAAG-3’  5’-CTGAGTATGTTATACTCCTCAAGC-3’ |
| *SMR5* | 5’-TTGCCGGATACCAGCATAC-3’  5’-GCGGCTGAAAATATCCCTTC-3’ |
| *SMR7* | 5’-CCCACCGGTGTTGAAATG-3’  5’-CGTTGTATAAACACCAACTCGAA-3’ |
| *MYB3R3* | 5’-TAAGGTCTTACCCGGCAGGACTGAT-3’  5’-CTGATGAGGTTTGAGCAACTGAACC-3’ |
| *MYB3R4* | 5’-AATCGCTTGAGAAAGTAGACC-3’  5’-AGTAGACAGGACTGGCTTACCG-3’ |

**ChIP-qPCR**

| *Mul* | 5’-GATTTACAAGGAATCTGTTGGTGGT-3’  5’-CATAACATAGGTTTAGAGCATCTGC-3’ |
| --- | --- |
| *ANAC044* | 5’-CTCGAAGCTTAGGGTCGGAA-3’  5’-TGGAGTGAACGCGTGAAGAC-3’ |
| *ANAC085* | 5’-ACGTTAGCCAGTTGTGTTCTTG-3’  5’-ACGTGTGTGTTGATGAGCGG-3’ |

**Semi-quantitative RT-PCR**

| *ACTIN2* | 5’-CTGGATCGGTGGTTCCATTC-3’  5’-CCTGGACCTGCCTCATCATAC-3’ |
| --- | --- |
| *ANAC044* | 5’-GAGCGCTAGAAAGGGAACGA-3’  5’-GCTTCCATGCTTTCGTCGC-3’ |
| *ANAC085* | 5’-AGCTAAGTGTGGTATTGATGGC-3’  5’-CTTCAATAACACTCACATTCCC-3’ |
